# Object Detection for Agricultural Vehicles: Ensemble Method Based on Hierarchy of Classes

**DOI:** 10.3390/s23167285

**Published:** 2023-08-20

**Authors:** Esma Mujkic, Martin P. Christiansen, Ole Ravn

**Affiliations:** 1 Automation and Control Group, Department of Electrical and Photonics Engineering, Technical University of Denmark, 2800 Kongens Lyngby, Denmark; or@elektro.dtu.dk; 2 AGCO A/S, 8930 Randers, Denmark; martinpeter.christiansen@agcocorp.com

**Keywords:** object detection, ensemble methods, agricultural vehicles

## Abstract

Vision-based object detection is essential for safe and efficient field operation for autonomous agricultural vehicles. However, one of the challenges in transferring state-of-the-art object detectors to the agricultural domain is the limited availability of labeled datasets. This paper seeks to address this challenge by utilizing two object detection models based on YOLOv5, one pre-trained on a large-scale dataset for detecting general classes of objects and one trained to detect a smaller number of agriculture-specific classes. To combine the detections of the models at inference, we propose an ensemble module based on a hierarchical structure of classes. Results show that applying the proposed ensemble module increases mAP@.5 from 0.575 to 0.65 on the test dataset and reduces the misclassification of similar classes detected by different models. Furthermore, by translating detections from base classes to a higher level in the class hierarchy, we can increase the overall mAP@.5 to 0.701 at the cost of reducing class granularity.

## 1. Introduction

Technological innovation is transforming the agricultural industry in order to meet increasing food demands and minimize environmental impact and sustainability [1,2]. Therefore, autonomous vehicles will be an essential part of future farming solutions. However, the currently available autonomous vehicles in agriculture cannot process the surrounding environment fully and safely perform complex field tasks without human intervention. Moreover, the environment in which those vehicles operate is highly unstructured and changes throughout different cycles. Therefore, the safe field operation of autonomous agricultural vehicles depends on a robust perception system and reliable visual data processing.

Object detection is an important part of scene understanding and obstacle avoidance for autonomous vehicles. The object detectors enable localization and classification of predefined classes of objects commonly found in the vehicle’s environment. The output provided by the object detector can later be used to either provide assistance to the vehicle operator or be integrated with the decision-making module in more autonomous solutions.

In recent years, due to the development of deep learning models and increased computational power, object detection has been applied in solving a wide range of real-life tasks [3]. In the agricultural domain, deep learning-based object detection has been used in fruit detection [4,5,6,7], remote sensing [8,9,10] and weed detection [11,12,13]. One of the main challenges in applying state-of-the-art object detectors for obstacle detection in agriculture is the availability of large-scale image datasets. Some of the public benchmark datasets for object detection, such as Microsoft Common Objects in COntext (MS COCO) [14] and PASCAL Visual Object Classes (VOC) [15], contain classes of objects like humans and some types of animals that are relevant for the agricultural domain as well. However, none of the benchmark datasets were collected for agricultural application and, therefore, lack the important agriculture-specific classes of objects such as different agricultural vehicles, implements, static objects commonly found in the fields, etc. Therefore, the previous research on deep learning-based obstacle detection in agriculture focused on detecting only a limited number of classes and does not include important domain-specific classes. Kragh et al. [16] applied two detection algorithms, LDCF and YOLO. Both algorithms are trained on publicly available datasets that are not intended for agriculture, and, therefore, the classes are limited to general domain classes such as ‘human’, ‘vehicle’ and ‘unknown’. Steen et al. [17] presented an algorithm for obstacle detection in agricultural fields based on AlexNet. The network is trained for the detection of ISO-standardized barrel-shaped obstacles. The presented algorithm performed well in detecting this type of obstacle but failed to detect other types of obstacles, highlighting the limitations of a standardized obstacle in image-based detection. Work by Li et al. [18] proposed a method for the detection of typical obstacles in orchards, such as humans, cement columns and utility poles. The method is based on YOLOv3 [19] and uses a lightweight MobileNetV2 network and Gaussian model to improve detections. In work by [20], four cameras were attached to the tractor to detect human presence during the tractor’s operation. The detection network is YOLOv3 trained on images of humans extracted from the MS COCO dataset.

**Figure 1:**
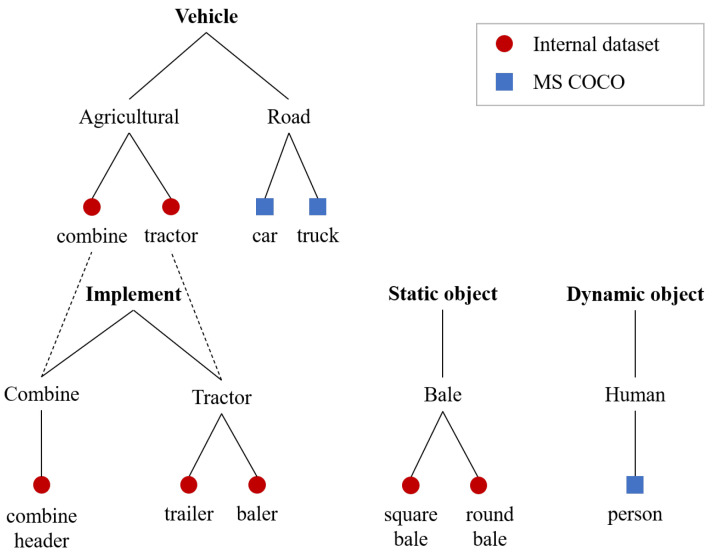
Hierarchy of classes in the dataset.

This paper focuses on the detection of objects in the agricultural vehicle’s environment during summer harvest operations. To address the challenge of limited dataset availability for the agricultural domain and leverage the benefits of already available models pre-trained on large-scale datasets able to detect classes from the general domain, we employed two detection models. The first model is a pre-trained model on MS COCO datasets with enabled detection of three classes—‘person’, ‘car’ and ‘truck’—selected as relevant for this scenario. The second model is trained on a smaller agriculture-specific dataset and is trained to detect seven classes: ‘tractor’, ‘combine’, ‘trailer’, ‘combine header’, ‘baler’, ‘square bale’ and ‘round bale’. Both models are based on the YOLOv5 [21] architecture. We observed that combining the detection results from both models by simple concatenation leads to redundant detection of agricultural vehicles as road vehicle classes. These two groups of classes share similar visual appearances and belong to the same higher-level category, ‘Vehicle’, as illustrated in the class hierarchy shown in Figure 1.

Existing ensemble methods that combine the output of detection models by eliminating redundant bounding boxes, such as those based on non-maximum suppression [22,23] or fusion [24], do not consider the class of the detected object. The ensemble approach proposed by Casado-García and Heras [25] considers the classes of objects by grouping the detected objects of the same class based on the intersection over union (IoU) threshold and applying one of the proposed three voting strategies for ensembling. In our case, the bounding boxes that need to be processed by the ensemble method belong to different classes, and the appropriate ensembling method needs to reflect the semantic relationships between their classes proposed in the class hierarchy.

Several approaches to object detection have exploited contextual relationships between the classes in order to improve detection performance. The work presented in [26] supplemented a pre-trained object detection model with a graph convolutional network (GCN). The GCN processes a relationship knowledge graph with the conditional probability generated for MS COCO classes. The reported results for the MS COCO dataset show that including GCN improves mean average precision. The super-class guided network (SGNet), proposed by Li et al. [27], improves the performance of image classification and object detection tasks by incorporating a two-level hierarchy of classes. The architecture comprises two branches, a super-class branch (SCB) and finer class branch (FCB), that operate in parallel. The SCB focuses on capturing general features shared by super-class categories, while the FCB handles detailed fine-grain attributes. During training, both branches are trained together, and the total loss used for backpropagation is calculated as the sum of losses in individual branches. In this way, misclassifications at the super-class level result in higher loss and affect parameter updates for FCB as well. The results show that the proposed SGNet improves performance on the classification task on CIFAR-100 dataset and object detection task on MS COCO datasets. The work presented in [28] addresses the training of object detectors on multiple datasets that have labels at different hierarchical levels. Single Shot multibox Detector: Multi-Loss (SSD-ML) is proposed as a modification of the SSD detector. The classification is decoupled from localization, and the binary loss function is used to generate prediction scores for each category independently. The proposed method outperforms the original SSD on traffic surveillance datasets. Salakhutdinov et al. [29] aimed to improve detection accuracy for classes with limited training data by sharing feature representations from related classes with more training data. The hierarchical Gaussian prior model (with a two-level prior) was used to represent relationships between classes of visually similar objects. In all of these works, the focus was on modifying the architectures to incorporate hierarchical relationships between classes during the training stage and improve the detection performance of a single model.

We propose an ensemble module that incorporates relationships from the hierarchy of classes at inference time and requires no training. Compared to our previous work [30], which focused on combining an object detector for agriculture-specific classes and an anomaly detector for detecting remaining potential obstacles, this work focuses on combining two object detectors, one for agriculture-specific classes and one for general domain classes, by taking into account the class hierarchy of the base classes that they detect. The two object detectors and the ensemble method proposed in this paper can be potentially used in place of the object detector in the framework proposed in our previous work. The proposed ensemble module consists of a set of rules for removing redundant detections for the case of agricultural and road vehicles and is not dependent on the architecture of the object detection models that are being ensembled. The performance of models is evaluated on a test dataset containing ten classes of objects that the two models are able to detect when combined. We show that applying the proposed ensemble module improves the mean average precision (mAP) on the test dataset. Moreover, we show that translating the model predictions to a higher level in the hierarchical structure of classes improves mAP even further at the cost of class granularity.

The paper’s main contributions are summarized below:

- We propose the class hierarchy for object detection in agriculture. The class hierarchy contains agriculture-specific classes as well as relevant classes from the publicly available dataset;
- We evaluate the performance of a model trained for agriculture-specific classes and a model pre-trained on MS COCO on an agricultural test dataset;
- We present a method for ensembling object detectors based on the proposed hierarchy of classes. The presented method ensembles the detections at inference time and is independent of underlying detection algorithms. The performance of combined models with and without the ensemble module is evaluated, and we show that the overall performance improves with the addition of the proposed ensemble module;
- We show that translating the model predictions to a higher level in the hierarchy can improve the performance even further at the loss of class granularity.

The remainder of the paper is structured as follows. Section 2 describes datasets, model architecture and ensemble module. In Section 3, the performance of the trained networks and the proposed ensemble module are evaluated. The conclusion follows in Section 4.

## 2. Methods

### 2.1. Datasets

This section describes the datasets used for the training and testing of the models.

#### 2.1.1. Common Objects in Context (COCO) Dataset

MS COCO [14] is a large-scale benchmark dataset for object recognition. The dataset contains 123k training and validation images, annotated for 80 classes of objects. The images are taken from everyday scenes containing common objects. As shown in Table 1, annotated classes of objects are grouped into 12 supercategories. Among the supercategories and classes, we identified the ones that could be relevant in the agricultural domain. Specifically, in the case of object recognition for agricultural vehicles, supercategories of persons, vehicles and animals are of interest. For this paper, the classes ‘person’, ‘car’ and ‘truck’ were selected. During field operations, it is very common for farmers and workers to be present in the field, especially around the vehicles during servicing. In addition, vehicles such as cars are often left parked at the field’s boundaries, and trucks are often used for unloading harvested grain. While animals can often interfere with field operations, especially wild animals, consideration of these classes is beyond the scope of this paper.

#### 2.1.2. Agricultural Dataset for Object Detection

The dataset used for training the model for object detection in an agricultural environment consists of 14,318 images annotated for seven agriculture-specific classes. The annotated classes are ‘tractor’, ‘combine’, ‘trailer’, ‘combine header’, ‘baler’, ‘square bale’ and ‘round bale’.

#### 2.1.3. Testing Dataset

For the testing of models, a dataset consisting of 7.9k images was collected by 2 agricultural vehicles over 13 days. The annotated classes are ‘tractor’, ‘combine’, ‘trailer’, ‘combine header’, ‘baler’, ‘square bale’, ‘round bale’, ‘person’, ‘car’ and ‘truck’. By adopting the hierarchical approach, the base classes are grouped into categories at two levels of granularity, as shown in Table 2. The first level, ‘subcategory’, represents coarse labels for the base classes. At the top level, labeled ‘supercategory’, the subcategories are grouped even further into more general categories such as ‘Vehicle’, ‘Implement’, ‘Static object’ and ‘Dynamic object’.

### 2.2. Object Detection Models

Two object detection models based on YOLOv5 were used for the detecting classes in the test dataset. One model is trained to detect seven agriculture-specific classes using transfer learning. The other model is a publicly available model trained on the MS COCO dataset.

The YOLOv5 models are single-stage object detectors consisting of a backbone network, neck and detection head. The cross-stage partial connections (CSP) network is used as a backbone that extracts features from the input image. In general, the neck of the network generates feature pyramids and enables the model to perform better when detecting objects of various sizes and scales. In YOLOv5, PANet is used as the neck network. The final detection is performed by the YOLO head proposed in YOLOv3 [19]. It generates the final output vector with class probabilities, objectness scores and bounding boxes from anchor boxes applied to features.

The model for the detection of agricultural classes is the YOLOv5m model trained on the agricultural dataset described in Section 2.1.2. The dataset was randomly split into training and validation datasets with a 70:30 ratio. The images were resized to 640 × 640, and the model was trained for 300 epochs using default hyperparameters.

The model used for detecting classes ‘person’, ‘car’ and ‘truck’ is the YOLOv5l model from Ultralytics [21], trained on the MS COCO dataset to detect 80 classes of common objects. During the non-max suppression stage, the three classes are selected from the predictions.

### 2.3. Ensemble Module

The pre-trained COCO model is able to detect ‘truck’ and ‘car’ classes of road vehicles, while the model for agricultural classes is able to detect classes of agricultural vehicles ‘combine’ and ‘tractor’. Since objects belonging to these classes have similar visual properties, the pre-trained model that has not been trained for detection in images depicting agricultural scenes specifically often detects agricultural vehicles as ‘truck’ or ‘car’. While implements do not belong to the ‘Vehicle’ super-category directly, they still share a lot of common properties with vehicles, such as having wheels, being made of similar material, having similar colour, etc. Therefore, they are also often detected as road vehicles by the pre-trained network. In the cases where both models detect the same object, the redundant road vehicle detections are removed if they exceed the overlap threshold with detections of agricultural classes.

Moreover, the classes for agricultural implements are closely linked with classes for agricultural vehicles. During field operation, agricultural vehicles often have an implement attached at the front or the back of the vehicles. This linkage between agricultural vehicles and implements creates a special case for the ensembling of detections. While the pre-trained model is good at detecting vehicles at least at the higher super-category level, it does not have the notion of the linkage between the agricultural vehicles and the implements classes. For example, consider a tractor with a trailer attached at the back. In this case, the model for agricultural classes will detect two objects, ‘tractor’ and ‘trailer’, while the pre-trained model will often detect these two objects as a single vehicle object ‘truck’. Therefore, these vehicle–implement pairs—in our case, tractor with trailer or baler attached or combine with combine header attached—need to be treated separately. In the cases where the agricultural model detects a vehicle–implement pair, the redundant truck detection that overlaps with the vehicle, implement or vehicle–implement pair detection is removed if their overlap exceeds a threshold. This threshold is lowered compared to the cases of redundant road vehicle detection for vehicles and implements that are not detected as being a pair.

The summary of rules applied in ensemble module and specific thresholds are provided below.

Detections of class ‘truck’ are removed based on IoU with the detections of agriculture-specific classes in the following cases:

- Agricultural vehicle paired with its implement: if IoU≥0.6;
- Individual vehicle and implement detections:
  - Part of vehicles and implement pair: if IoU≥0.6;
  - Not part of vehicle and implement pair: if IoU≥0.8.

Detections of class ‘car’ are removed based on IoU with the detections of agriculture-specific classes in the following cases:

- Individual vehicle and implement detections: if IoU≥0.8.

**Figure 2:**
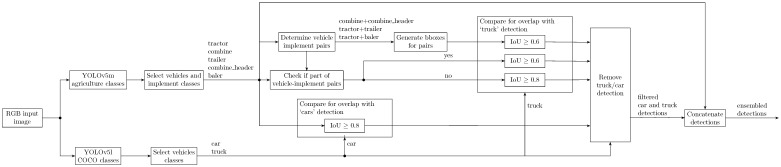
Diagram of the ensemble module.

The diagram of the ensemble module is provided in Figure 2. First, the image is passed through both detection models and detection results are obtained. The detections for classes belonging to agricultural vehicles and implements are processed to determine which detections form vehicle–implement pairs. Then, for every vehicle–implement pair, a bounding box for the vehicle object and a bounding box for the implement object are merged to form a new bounding box around the vehicle–implement pair. Additionally, all vehicle and implement detections that are a part of the vehicle–implement pair are flagged. The bounding boxes corresponding to vehicle–implement pairs are compared for overlap with detections of class ‘truck’ coming from the pre-trained model. If the overlap exceeds a certain threshold, the ‘truck’ detection is removed. The individual vehicle and implement detections that have been determined as part of the vehicle–implement pair are also compared with ‘truck’ detections in the same way, and ‘truck’ detections are removed based on the overlap threshold. Individual vehicle and implement detections that are not part of vehicle–implement pairs are also compared to ‘truck’ detection, but in this case, the overlap threshold for removal of ‘truck’ is higher. When it comes to the class ‘car’, the detections are compared with all detections belonging to agricultural vehicles or implements and removed if the overlap exceeds the thresholds. Finally, the remaining ‘truck’ and ‘car’ detections are concatenated with detections of agricultural classes.

## 3. Results and Discussion

This section analyzes the performance of the models in three cases. First, the performance of the individual models on the test dataset is evaluated. Then, the performance of both models combined with the ensemble module is evaluated. Finally, the performance of the models at the subcategory level is evaluated.

### 3.1. Performance Evaluation of Individual Models

First, the performance of the individual models on the test dataset was evaluated. As expected, the models were not able to detect the classes in which they were not trained. This results in an mAP equal to zero for these classes, affecting the average mAP on the test dataset. The results are shown in Table 3.

The model trained on the internal dataset has good mAP for all the classes it has been trained on, except ‘baler’. However, because the model is not able to detect classes ‘person’, ‘car’ and ‘truck’, the average mAP@.5 for all classes is 0.409, and mAP@.5:.95 is 0.277.

The model trained on the MS COCO dataset is able to detect classes ‘person’ and ‘car’ very well, while class ‘truck’ has low mAP. However, similar to the internal model, the mAP is affected by the classes the model cannot detect, resulting in mAP@.5 equal to 0.166 and mAP@.5:.95 equal to 0.112.

It can be concluded from the presented results that the individual models themselves do not perform adequately on the test dataset.

### 3.2. Performance with the Ensemble Module

The performance of combined models was evaluated with the ensemble module and compared to the performance without the ensemble module. The results are reported using a multiclassification confusion matrix and mAP at an IoU threshold equal to 0.5 as well as averaged over 10 IoU thresholds [0.5:0.95].

First, the detections of the two models were concatenated into a combined output without the ensemble module, and the performance was evaluated. Compared to the performance of the individual models, the combined model is able to detect all classes in the test dataset. Therefore, the overall performance computed as mAP@.5 increased from 0.49 and 0.166 for individual models to a combined mAP@.5 of 0.575, shown in Table 4.

Then, the ensemble module is added and applied to the combined output, and the performance is evaluated with emphasis on the misclassification of similar classes detected by the two models.

**Figure 3:**
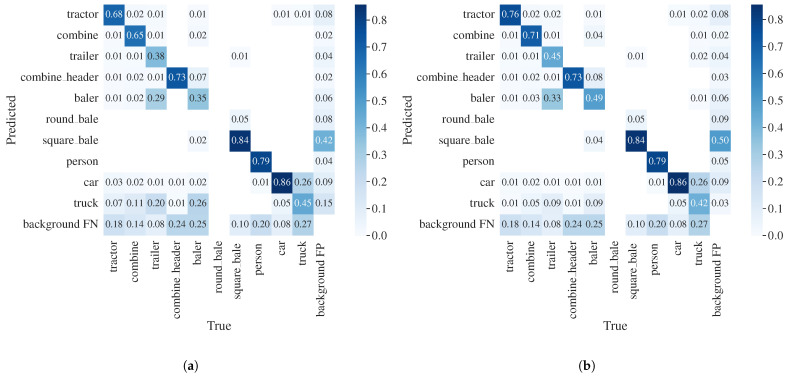
Confusion matrix. (**a**) Combined detections without ensemble module. (**b**) Combined detections with ensemble module.

A confusion matrix comprehensively illustrates the classification accuracy of object detectors as well as the misclassification rate for pairs of classes. The confusion matrices for the combined model without the ensemble module and with the ensemble module are shown in Figure 3. The rows in the matrix correspond to the instances predicted to belong to a class, and the columns correspond to the instances in the actual class. In the calculation of the confusion matrix, only boxes with a confidence score greater than 0.25 are considered. The IoU threshold for ground truth and detected bounding box is set to 0.45, and columns in the confusion matrices are normalized.

It can be seen that the ensemble module has significantly better performance, and the confusion between road vehicle classes and classes for agricultural vehicles and implements is lower. Initially, in the combined model, the classes ‘trailer’ and ‘baler’ are most often confused with class ‘truck’. However, after applying the ensemble module, the values of corresponding elements in the confusion matrix are lower and values in diagonal elements for classes ‘trailer’ and ‘baler’ improved. A similar trend can be observed for classes ‘tractor’ and ‘combine’, while values for ‘combine header’ remain the same. When it comes to the class ‘car’, there is a small decrease in the misclassification of classes ‘tractor’ and ‘baler’.

Considering the results in Table 4, it can be seen that the overall mAP@.5 increased from 0.575 to 0.605 when the ensemble module was applied. The same can be observed with mAP@.5:.95, which increased from 0.39 to 0.404. The increase is mostly due to the increase in mAP for class ‘truck’. This resulted from the removal of the misclassification of agricultural vehicles and implements as ‘truck’, as these are considered false positives for this class.

**Figure 4:**
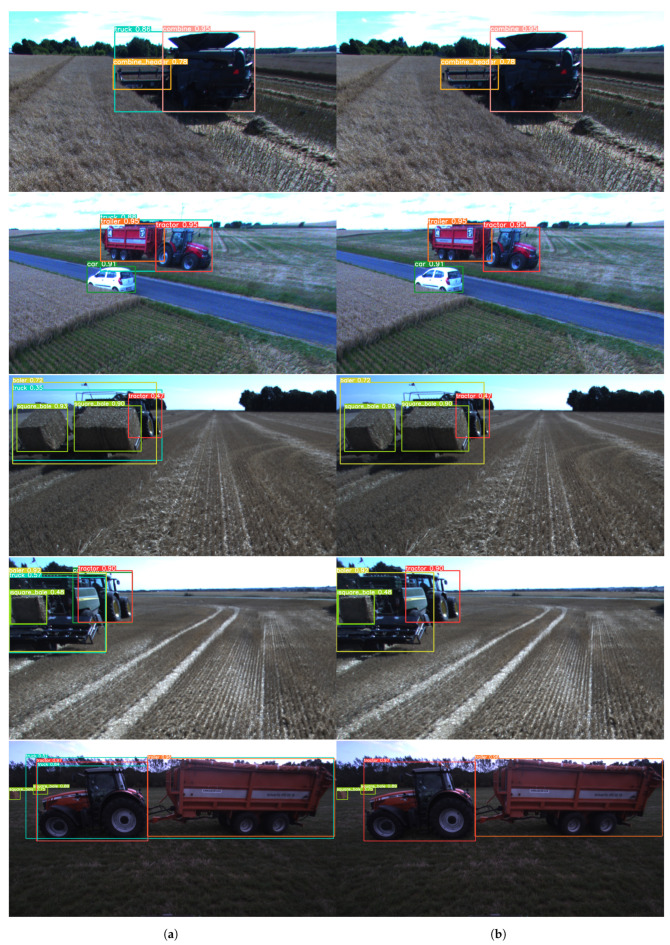
Detection examples. (**a**) Combined models without ensemble module. (**b**) Combined models with ensemble module.

The qualitative performance of the ensemble module is presented in Figure 4. The first example shows the ‘truck’ detection removed because the internal model detected the ‘combine’ and ‘combine header’ pair. The second example shows ‘truck’ detection removed because the internal model detected the ‘tractor’ and ‘trailer’ pair. The third example shows a ‘baler’ attached to a ‘tractor’ and two ‘square bales’. In this case, the redundant ‘truck’ detection was also removed by the ensemble module successfully. The fourth example again shows a ‘baler’ attached to a ‘tractor’ and one ‘square bale’. In this case, there are two redundant detections, one ‘truck’ detection corresponding to ‘baler’ and one ‘car’ detection corresponding to ‘tractor’. The ensemble module removes both redundant detections. The fifth example shows a ‘trailer’ attached to a ‘tractor’ and two redundant ‘truck’ detections. One detection corresponds to the ‘tractor’ and ‘trailer’ pair and the other to the ‘tractor’ only. Both are removed successfully by the ensemble module.

The chosen baseline method, without the ensemble module, is equivalent to combining the predictions of the two models and applying non-maximum suppression (NMS). Since non-maximum suppression is applied per class to remove redundant detections, it does not address redundancy across the classes, as is the case with classes belonging to agricultural vehicles and road vehicles. A modification of NMS, agnostic NMS, performs non-maximum suppression across all classes simultaneously, and the resulting detections are selected irrespective of their class labels. This approach makes it possible to remove redundant detections of the same object instance which might have been predicted as different classes. Therefore, it was applied for comparison, and the results are presented in Table 5.

While applying agnostic NMS shows improvements in mAP@.5 and mAP@.5:.95 for classes ‘truck’, ‘combine’, ‘combine_header’ and ‘baler’ for classes ‘trailer’ and ‘square_bale’, the performance is decreased. Therefore, with the overall mAP@.5 0.557 and mAP@.5:.95 of 0.379, agnostic NMS performs worse than baseline NMS and the proposed ensemble module. This can be explained by the relatively high confidence scores of detections from a model trained on MS COCO, even for the object instances that belong to the agricultural domain. Moreover, the agnostic NMS takes into account individual detections only and not the detections of vehicle–implement pairs when removing redundant road vehicle detections as the proposed ensemble module does.

### 3.3. Performance Evaluation at Subcategory Level

Considering the hierarchy of classes shown in Figure 1, the performance of the models with the proposed ensemble module was evaluated at the subcategory level. Base class detections were translated into 6 subcategories: ‘agricultural vehicle’, ‘tractor implement’, ‘combine implement’, ‘bale’, ‘road vehicle’ and ‘human’. The results are again reported using a confusion matrix and mAP.

**Figure 5:**
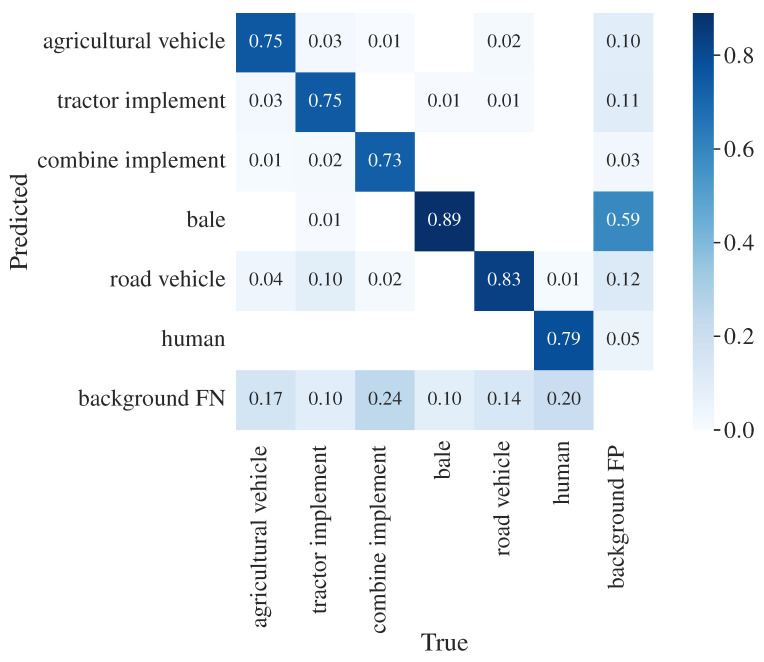
Confusion matrix for subcategory detection with ensemble module.

The confusion matrix in Figure 5 shows that the model performs well when evaluated at the subcategory level. The highest misclassification is between ‘road vehicle’ and ‘tractor implement’. This is expected since the misclassification of classes ‘trailer’ and ‘baler’ with class ‘truck’ was high at the base class level. Moreover, the high misclassification between base classes in the subcategories ‘tractor implement’, between ‘trailer’ and ‘baler’, and in subcategory ‘road vehicle’, between ‘car’ and ‘truck’, does not affect the performance at the subcategory level.

The results in Table 6 indicate that the overall detection performance improves at the subcategory level. This is due to the previously mentioned misclassification between base classes in the same subcategories, which is removed once the base classes are translated to subcategories.

### 3.4. Application Prospects

The proposed object detection approach has great application potential in the automation of different agricultural tasks. One example is detecting the cooperative machine within the agricultural machine’s environment and performing a cooperative operational task. The application use cases can be the detection of unloading vehicles for combine harvesters and forage harvesters or the detection of leader/follower vehicles. Another potential application is the coordination of the operation of multiple agricultural machines within the same working environment through vehicle tracking.

Moreover, the proposed hierarchy of classes that the ensemble method is based on facilitates altering class granularity by mapping classes to higher-level categories. This enables customizing classes of trained models for tasks that do not require a high level of class granularity.

## 4. Conclusions

This work presented an ensemble method for object detectors in agriculture based on the hierarchical structure of classes. The proposed hierarchy includes agriculture-specific and general domain classes and highlights the linkage between them. Two YOLOv5 models were used to detect different classes within an agricultural image dataset. The results showed that the models did not perform well individually on the dataset. However, combining their detections increased mAP significantly. Moreover, applying the proposed ensemble module to combined detections from the two models further improved mAP@0.5 from 0.575 to 0.605. Finally, translating classes of detected objects to a higher level in the class hierarchy demonstrated that it is possible to increase mAP@0.5 to 0.701 at the cost of class granularity.

Future work will investigate the possibilities of including more relevant classes in the hierarchy and exploring ensemble strategies at the subcategory and supercategory levels. 

## Figures and Tables

**Table 1:** Overview of classes in the MS COCO dataset.

Supecategory	Classes
**Person** ^1^	**person**
**Vehicle**	bicycle, **car**, motorcycle, airplane, bus, train, **truck**, boat
Outdoor	traffic light, fire hydrant, stop sign, parking meter, bench
Animal	bird, cat, dog, horse, sheep, cow, elephant, bear, zebra, giraffe
Accessory	backpack, umbrella, handbag, tie, suitcase
Sports	frisbee, skis, snowboard, sports ball, kite, baseball bat, baseball glove, skateboard, surfboard, tennis racket
Kitchen	bottle, wine glass, cup, fork, knife, spoon, bowl
Food	banana, apple, sandwich, orange, broccoli, carrot, hot dog, pizza, donut, cake
Furniture	chair, couch, potted plant, bed, dining table, toilet
Electronic	tv, laptop, mouse, remote, keyboard, cell phone
Appliance	microwave, oven, toaster, sink, refrigerator
Indoor	book, clock, vase, scissors, teddy bear, hair drier, toothbrush

^1^ Bold denotes classes and corresponding supercategories selected for this paper.

**Table 2:** Overview of classes in the test dataset.

Supercategory	Subcategory	Class
Vehicle	Agricultural	tractor
		combine
	Road	car
		truck
Implement	Tractor	trailer
		baler
	Combine	combine header
Static object	Bale	square bale
		round bale
Dynamic object	Person	person

**Table 3:** Performance of individual models on the test dataset.

Class	Internal Model		COCO Model	
	mAP@.5	mAP@.5:.95	mAP@.5	mAP@.5:.95
tractor	0.731	0.536	0	0
combine	0.689	0.497	0	0
trailer	0.655	0.459	0	0
combine_header	0.672	0.399	0	0
baler	0.0788	0.0338	0	0
square_bale	0.858	0.571	0	0
person	0	0	0.649	0.39
car	0	0	0.778	0.59
truck	0	0	0.065	0.0308
average	0.409	0.277	0.166	0.112

**Table 4:** Comparison of combined detections of two models without ensemble module and with the ensemble module.

Class	Without Ensemble Module		With Ensemble Module	
	mAP@.5	mAP@.5:.95	mAP@.5	mAP@.5:.95
tractor	0.731	0.536	0.731	0.536
combine	0.689	0.497	0.689	0.497
trailer	0.655	0.459	0.655	0.459
combine_header	0.673	0.399	0.672	0.399
baler	0.0787	0.0338	0.0787	0.0338
square_bale	0.858	0.571	0.858	0.571
person	0.649	0.39	0.649	0.39
car	0.777	0.59	0.725	0.549
truck	0.065	0.0308	0.386	0.201
average	0.575	0.39	0.605	0.404

**Table 5:** Perfromance of agnostic non-maximum suppression.

Class	Agnostic NMS	
	mAP@.5	mAP@.5:.95
tractor	0.731	0.54
combine	0.698	0.518
trailer	0.472	0.338
combine_header	0.688	0.411
baler	0.0865	0.0376
square_bale	0.825	0.552
person	0.648	0.391
car	0.777	0.588
truck	0.0863	0.0396
average	0.557	0.379

**Table 6:** Performance of the models with ensemble module translated to subcategory level.

Class	With Ensemble Module	
	mAP@.5	mAP@.5:.95
agricultural vehicle	0.697	0.51
tractor implement	0.688	0.466
combine implement	0.672	0.399
bale	0.86	0.571
road vehicle	0.64	0.461
human	0.649	0.39
average	0.701	0.466

## Data Availability

The data presented in this study are available on request from the corresponding author.
